# Identification of hip fracture patients from radiographs using Fourier analysis of the trabecular structure: a cross-sectional study

**DOI:** 10.1186/1471-2342-4-4

**Published:** 2004-10-06

**Authors:** Jennifer S Gregory, Alison Stewart, Peter E Undrill, David M Reid, Richard M Aspden

**Affiliations:** 1 Department of Orthopaedics, University of Aberdeen, Aberdeen, United Kingdom; 2 Department of Medicine and Therapeutics, University of Aberdeen, Aberdeen, United Kingdom; 3 Department of Biomedical Physics and Bioengineering, University of Aberdeen, Aberdeen, United Kingdom

## Abstract

**Background:**

This study presents an analysis of trabecular bone structure in standard radiographs using Fourier transforms and principal components analysis (PCA) to identify contributions to hip fracture risk.

**Methods:**

Radiographs were obtained from 26 hip fracture patients and 24 controls. They were digitised and five regions of interest (ROI) were identified from the femoral head and neck for analysis. The power spectrum was obtained from the Fourier transform of each region and three profiles were produced; a circular profile and profiles parallel and perpendicular to the preferred orientation of the trabeculae. PCA was used to generate a score from each profile, which we hypothesised could be used to discriminate between the fracture and control groups. The fractal dimension was also calculated for comparison. The area under the receiver operating characteristic curve (*A*_*z*_) discriminating the hip fracture cases from controls was calculated for each analysis.

**Results:**

Texture analysis of standard radiographs using the fast Fourier transform yielded variables that were significantly associated with fracture and not significantly correlated with age, body mass index or femoral neck bone mineral density. The anisotropy of the trabecular structure was important; both the perpendicular and circular profiles were significantly better than the parallel-profile (*P *< 0.05). No significant differences resulted from using the various ROI within the proximal femur. For the best three groupings of profile (circular, parallel or perpendicular), method (PCA or fractal) and ROI (*A*_*z *_= 0.84 – 0.93), there were no significant correlations with femoral neck bone mineral density, age, or body mass index. PCA analysis was found to perform better than fractal analysis (*P *= 0.019).

**Conclusions:**

Both PCA and fractal analysis of the FFT data could discriminate successfully between the fracture and control groups, although PCA was significantly stronger than fractal dimension. This method appears to provide a powerful tool for the assessment of bone structure in vivo with advantages over standard fractal methods.

## Background

The NIH Consensus Statement defines Osteoporosis as "a skeletal disorder characterised by compromised bone strength predisposing to an increased risk of fracture" [1]. Bone strength was defined as "the integration of two main features: bone density and bone quality". Currently, clinical diagnosis is based solely on bone mineral density (BMD) in accordance with the World Health Organisation guidelines [2]. Previous studies, however, have found that trabecular bone structure also plays a significant role in determining bone strength [3-5] with BMD explaining only 60 to 80 % of the variability in mechanical resistance [6].

Trabecular bone structure is visible on standard pelvic radiographs and many attempts have been made to quantify the quality of the structure and assess its relationship to osteoporosis and BMD. These range from visual scoring systems, such as the Singh index [7], through to sophisticated computerised methods based on fractals [8-10] and other image processing methods [11-13]. A review of the literature suggests that fractal analysis has been a method of choice in recent years for the analysis of trabecular bone structure in CT scans [14,15], MRI [16], histology [17] and radiographs [18-21], although it has not been established categorically that it is preferable to other methods of texture analysis [22,23]. By reducing all the information in the image to one descriptor, the fractal dimension [24], a large part of the information is lost. The Fourier transform of an image expresses the information in the image in terms of spatial frequencies rather than distances. Various methods can be applied to extract information from the Fourier transform [25], including the fractal dimension [24]. However such methods have not been fully exploited for analysing bone structure [8,26-30].

In this study we investigate the use of Fourier transforms and Principal Components Analysis to generate a mathematical model of the data which can be used to help classify individuals according to the presence or absence of a hip fracture. Principal component analysis (PCA) [31] is a data reduction technique that has been applied in many fields of study, including investigation of gene expression [32], development of an electronic nose [33] and tracing of the evolutionary changes in fish morphometry [34]. It describes data in terms of a small number of orthogonal, linearly independent components which contain the majority of the information. PCA has no preconditions, such as relying on the data to fit a normal or fractal distribution, but builds a mathematical model based on the correlations present in the data. An eigenanalysis of the correlation or covariance matrix is used to perform PCA. The resulting components are then selected in order of the amount of variance they account for, enabling an efficient mapping of the data. As the first few components account for the vast majority of the variance in the original data, they can be selected for analysis whilst the remainder are discarded as 'noise'. In this way, the number of variables can be greatly reduced whilst maintaining the information present in the original data. In this pilot study we used these methods to investigate the similarities and differences between trabecular bone structure in fracture and control groups using standard radiographs of the proximal femur.

## Methods

### Study data

A set of digitised standard pelvic radiographs was available from a previous investigation into the morphology of the proximal femur [35]. These radiographs were taken from an earlier study [36], that had examined three groups (osteoporotic, osteoarthritic and control) of age matched, postmenopausal women (30 subjects per group). Subjects with osteoarthritis were excluded from the present study. All patients had undergone a scan of the unfractured hip by dual-energy x-ray absorptiometry (DXA) using a Norland XR-26 scanner (CooperSurgical Inc, Trumbull, CT). The controls had had their left hip scanned. All patients and controls had had a pelvic antero-posterior radiograph recorded within a year of the DXA scan. We used those radiographs and the femoral neck BMD (Neck-BMD) data in the current study. A data set of 50 digitised radiographs was available comprising 26 hip fracture patients (HIP) and 24 controls (CNT). The radiographs were digitised, using a Howtek MultiRAD 850 scanner (Howtek, Hudson, New Hampshire) at a resolution of 584 dpi (44 μm per pixel) and a depth of 12 bits. The age, height and weight of each subject were also recorded.

### Region selection

**Figure 1:**
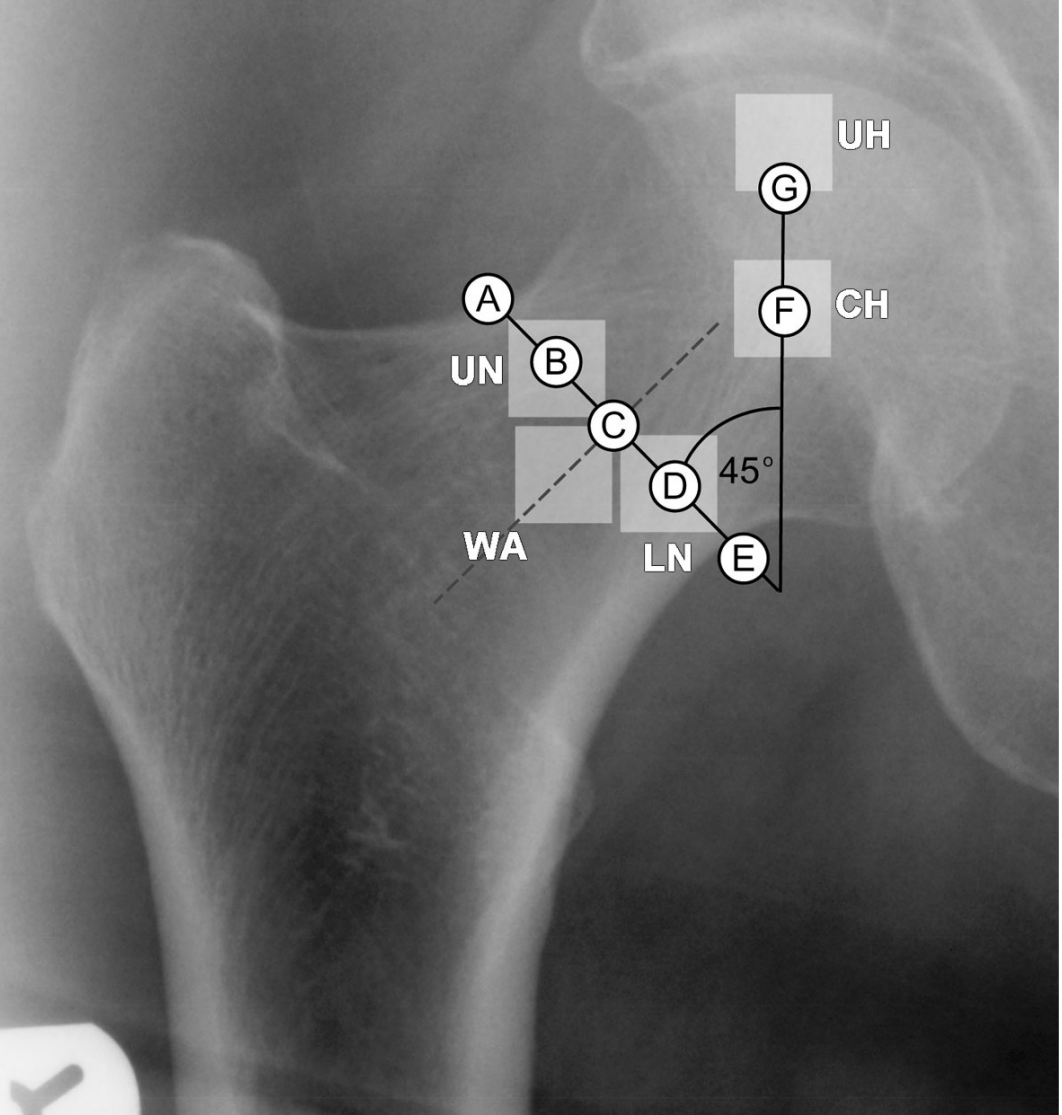
**Regions of interest. **Displays the five regions of interest, upper femoral head (UH), central femoral head (CH), upper femoral neck (UN), Ward's triangle area (WA) and the lower femoral neck (LN) used for analysis. Points A to G are determined by the femoral head and neck and used to locate the ROIs. Points A and E mark the femoral neck width. Points B, C and D lie at 1/4, 1/2 and 3/4 along this line. Point F is the centre point of the femoral head, point G at 1/2 the radius of the femoral head at an angle of 45 degrees to the neck width, 135 degrees to the neck shaft, shown as a dashed line through point C.

Five regions of interest (ROIs) were selected relative to the principal trabecular systems in locations known to be related to hip fracture via the Singh index [7] and BMD analysis [37]. To ensure reproducibility, their locations were determined in relation to the centre and angle of the narrowest part of the femoral neck and the centre and radius of the femoral head on each image, as shown in Figure 1.

Each ROI was 256 × 256 pixels (11.3 mm square), to enable use of the fast Fourier transform, and were selected as follows. The upper region of the head (UH) lies on the upper part of the principal compressive trabeculae, the central region of the head (CH) is at the intersection of the principal compressive and tensile trabeculae, the upper region of the neck (UN) lies on the principal tensile trabeculae, the lower region of the neck (LN) is at the base of the principal compressive trabeculae and finally Ward's triangle (WA) which lies between these structures. The points and regions were identified using a macro written for Image Pro Plus software (version 4.1.0.0, Media Cybernetics, Silver Spring, Maryland). The femoral head was described by a best-fit circle, calculated from a series of manually marked points around the outline of the femoral head. Between 15 and 20 evenly spaced points were used to describe the outline, depending on the size of the head. The radius and centre (marked as F in Figure 1) of the femoral head were then taken from this circle. The narrowest part of the neck (neck-width) was determined using two automatic edge traces, marking the upper and lower outlines of the femoral neck. The first point and the direction for each trace were marked manually; the edge of the neck could then be identified automatically by the software. The neck width (A – E in Figure 1) was calculated by finding the smallest Euclidean distance between the traces. The centre of the neck was located at the mid-point of this line (point C) and the axis of the femoral neck was taken to be a line perpendicular to this through the centre of the neck (dashed line). The top right corner of the WA region was located at the midpoint of the neck width (point C). Points B and D were placed 25% and 75% of the way along the neck width and used as the midpoints of the UN and LN regions respectively. Point F, the centre of the femoral head marked the centre of the CH region and point G, the centre of the base of the UH region. Point G was placed one half of the femoral head radius above point F, at a 45-degree angle to the neck width (A-E).

### Region analysis

Analysis was performed using Matlab software (version 6.1.0, MathWorks Inc, Natick, Massachusetts). A fast Fourier transform was generated for each ROI and three profiles were generated using data from the power spectrum. Firstly a global or circular profile (CircP) was generated, composed of the magnitude at each spatial frequency averaged across all angles, resulting in a profile with 128 data points. To create this profile, each pixel in the Fourier transform was assigned to the integer spatial frequency that most closely matched its' distance from the zero'th component.

**Figure 2:**
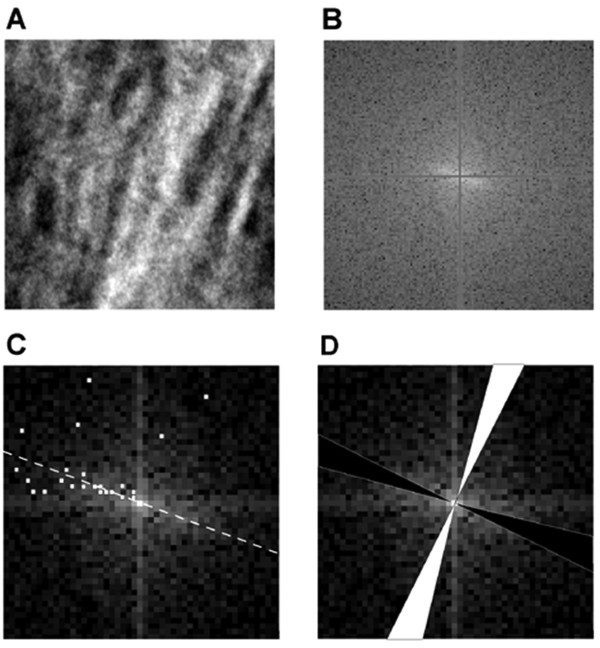
**Profile generation. **(A) Shows a typical region of interest (contrast enhanced for visualisation) showing the trabecular bone structure, in this case aligned approximately 22° to the vertical. (B) The central section of the FFT (128 × 128 pixels). The horizontal and vertical axes have been marked with a mid-grey tone to indicate that they have been excluded from the angle calculation. The bright strip at the centre (running from top left to bottom right) shows the preferred orientation of the trabeculae. Angles calculated from the Fourier power spectrum correspond to the same angles in the spatial domain, rotated by 90°. (C) The pixels with the maximum values are marked using white squares for the first 25 spatial frequency values of the Fourier power spectrum. The median angle, lying 21.8° from the horizontal is shown by a dashed white line. (D)_The regions used to generate the parallel (shaded black) and perpendicular (shaded white) profiles, based on the orientation of the trabecular structure.

The angle of preferred orientation was calculated by finding the angle of the maximum value in the power spectrum for the first 25 spatial frequencies [38]. The maximum value over this range relates to the dominant texture orientation within the image, the trabecular structure. As data in the frequency domain relate to features in the spatial domain rotated by 90°, the median of the values plus 90° was taken as the angle of preferred orientation for each image. Due to the symmetry of the Fourier power spectrum, angles were only calculated between 0° and 180°, rather than 0° and 360°. Two more profiles were then generated, parallel with (ParP) and perpendicular to (PerP) the angle of preferred orientation. In this case the average value was calculated at each spatial frequency from all points lying within ± 5° of the desired angle (Fig. 2).

### Principal component analysis

**Figure 3:**
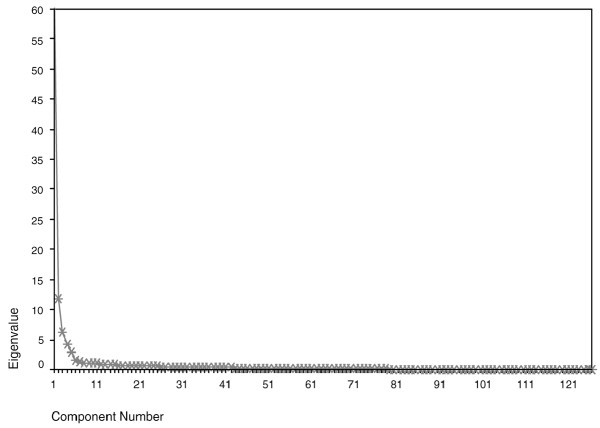
**Scree plot. **Example of a scree plot from the perpendicular profile. The first component typically accounts for the largest amount of variance. The components are chosen to the left of an 'elbow' in the plot. Here components 1 to 5 are included in the analysis as they lie before the 'elbow' at point6 (eigenvalue = 1.63).

Principal component analysis [31] was used to model statistically the shape of each set of profiles (parallel, perpendicular and circular). This was performed using an eigenanalysis of the correlation matrix. The eigenvectors then become the principal components and are selected in order, depending on their eigenvalue. The eigenvalues are associated with the components in decreasing order, the largest eigenvalue is associated with the first component and the smallest with the last. In order to choose the number of components for analysis, a scree plot [31,39] was generated by plotting the eigenvalues (representing the proportion of variance described by each component) against the component number (Figure 3). In each case, the first few principal components were selected for analysis using the scree test [39] to find an 'elbow' in the slope of the plot. This is used as a threshold between the components that contained useful information, which were then used as input variables for further analysis, and those that could be attributed to noise.

### Fractal analysis

Fractal analysis was performed on each profile using a method similar to the Fourier transform technique described by Majumdar et al [40]. The average power spectrum of the circular profile was plotted on a log-log scale, three approximately linear regions were defined and the gradient (*slope*) of a straight line fitted to each region was found; *slopeA*, a 'coarse' slope, where the log of the spatial frequency is less than or equal to 1.0, *slopeB *a 'medium' slope, where the log of the spatial frequency lies between 1.0 and 1.75 and *slopeC*, a 'fine' slope where the log of the spatial frequency is above 1.75. The fractal dimension was calculated for each slope using the formula suggested by Majumdar et al [40]



### Statistical analysis

Stepwise discriminant analysis was used to select principal components that could be combined to build a linear classifier. If the stepwise procedure failed to select any components, the most accurate of the individual components was chosen. The same procedure was used to discriminate between the groups using the fractal dimension. Measurement of the area under the ROC curve was used to compare the classifiers built using the discriminant analysis [41]. A three way ANOVA was applied in order to determine whether there were significant differences between the performance of classifiers depending on the type of analysis, the profile used or the region analysed. Pearson product moment correlation was applied to examine the relationship with age, BMI and Neck BMD for the strongest classifiers. A one-way ANOVA was used to test for significant differences in the performance of the slopes from each spatial frequency band used in the fractal analysis. T-tests, correlation and ANOVA were performed using SigmaStat (version 2.03, SPSS Science, Chicago). Principal component analysis, discriminant analysis, and measurement of the area under the ROC curve were calculated using SPSS (version 10 SPSS Science, Chicago).

## Results

There were no significant differences between the age, height, weight or body mass index (BMI) of the fracture and control groups (Table 1). As expected femoral neck-BMD was significantly lower in the fracture group in comparison to the control group (*P *= 0.001).

**Table 1:** Summary of anthropometric variables for the fracture and controls groups. Mean and standard deviation (SD) of the age, height, weight, BMI and BMD of the fracture and control groups. *P *values were obtained from a two-tailed t-test.

**Variable**	**Control Group (n = 24)**		**Fracture Group (n = 26)**		
	**Mean**	**SD**	**Mean**	**SD**	***P***
Age, years	69.1	6.5	69.2	6.3	0.97
Height, cm	158.6	7.1	157.1	0.4	0.38
Weight, kg	63.4	9.5	61.0	9.0	0.38
Body Mass Index, kg/m^2^	25.2	3.2	24.8	4.1	0.72
**Femoral neck BMD (g cm^-2^)**	**0.70**	**0.11**	**0.604**	**0.066**	**0.001**

**Figure 4:**
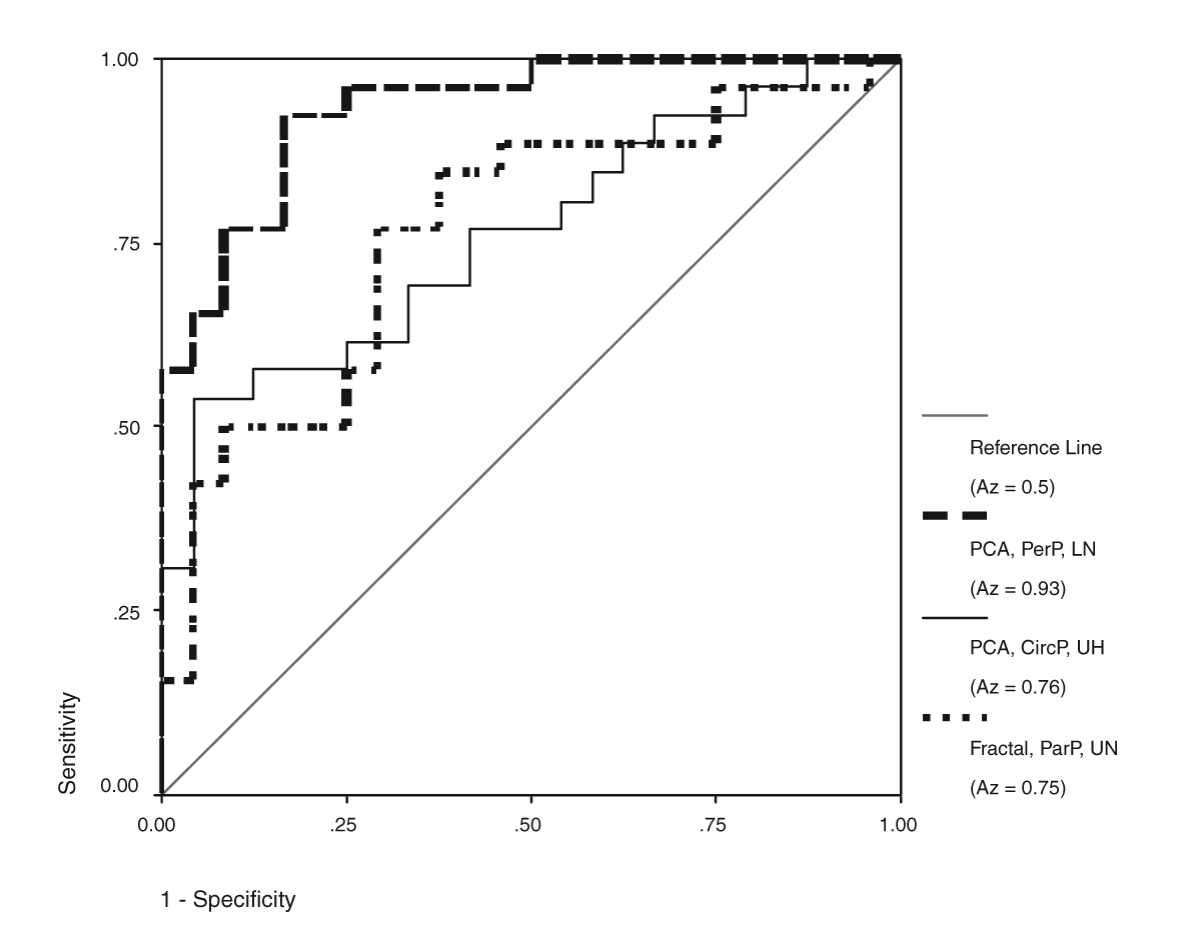
**Comparison of ROC curves. **Comparison of the ROC curves for the strongest classifier from the combination of (A) PCA analysis of the perpendicular profile (Lower neck region), (B) PCA analysis of the circular profile (Upper head region) and (C) Fractal analysis of any profile (Upper neck region).

The Receiver Operating Characteristic (ROC) curve is a plot of True Positive Fraction v False Positive Fraction (or Sensitivity v 1 – Specificity). The area underneath the curve (*A*_*z*_) represents the performance of the classifier ranging from a value of 0.5 if it is no better than chance to 1.0 for a perfect discriminator. Table 2 shows *A*_*z *_for PCA analysis by region for the circular, perpendicular and parallel profiles respectively, discriminating fracture and control cases. A wide range of values was observed (overall mean 0.70, standard deviation 0.11). Some were little better than chance (*A*_*z *_= 0.5) (mostly derived from the parallel profile) and the strongest ones were from the perpendicular profiles. The 5 largest areas under the ROC curve were obtained by PCA of the perpendicular profile of the lower neck, upper and central head regions (Table 3) (*A*_*z *_= 0.93, 0.84 and 0.84 respectively), followed by PCA analysis of the circular profile in the upper head region (*A*_*z *_= 0.76) and, finally, fractal analysis of the parallel profile in the upper neck region (*A*_*z *_= 0.75). Femoral neck BMD lay between the third and fourth best texture measures (*A*_*z *_= 0.79 95% CI = 0.66 – 0.91). Plots of the ROC curves for the strongest combinations of image analysis classifier are shown in Figure 4.

**Table 2:** Classification accuracy for each region-profile combination. Area under the ROC curve for principal component analysis of each profile by region of the femoral neck. Analysis using three-way ANOVA found that the area under the ROC curve was significantly higher in the perpendicular profile than in the parallel profile. (*P *< 0.05)

**Region**	**Circular (95% CI)**	**Parallel (95% CI)**	**Perpendicular (95% CI)**
Upper head	0.76 (0.63 – 0.89)	0.57 (0.41 – 0.73)	0.84 (0.73 – 0.95)
Central head	0.59 (0.43 – 0.75)	0.56 (0.40 – 0.73)	0.84 (0.72 – 0.95)
Upper neck	0.72 (0.58 – 0.86)	0.72 (0.57 – 0.86)	0.67 (0.52 – 0.82)
Wards triangle	0.74 (0.61 – 0.88)	0.61 (0.45 – 0.76)	0.71 (0.56 – 0.86)
Lower neck	0.71 (0.56 – 0.85)	0.55 (0.39 – 0.71)	0.93 (0.87 – 1.00)

**Table 3:** The best five classifiers: Area under the curve and correlation with BMD, age and BMI. Area under the ROC curve (*A*_*z*_) for each of the best 5 classifiers and the correlation with age R_age_, femoral neck BMD (R_BMD_) and body mass index (R_BMI_) and associated significance values (*P*).

**Analysis**	**Profile**	**ROI**	***A*_*z*_(95% CI)**	**R_BMD_(*P*)**	**R_age_(*P*)**	**R_BMI_(*P*)**
PCA	PerP	LN	0.93 (0.87 – 1.00)	0.09 (0.55)	0.14 (0.34)	-0.08 (0.58)
PCA	PerP	UH	0.84 (0.73 – 0.95)	0.09 (0.52)	-0.17 (0.24)	-0.03 (0.86)
PCA	PerP	CH	0.84 (0.72 – 0.95)	0.06 (0.70)	0.27 (0.055)	-0.11 (0.46)
PCA	CircP	UH	0.76 (0.63 – 0.89)	-0.16 (0.28)	-0.15 (0.29)	0.07 (0.62)
Fractal	ParP	UN	0.75 (0.61 – 0.89)	**-0.30 (0.034)**	0.25 (0.081)	-0.04 (0.78)

Table 3 also shows the correlations between the top five classifiers with age, BMI and Neck-BMD. No significant correlations were found between any of these classifiers and either age or BMI and, for the top three, there was also no significant correlation with Neck-BMD (*P *> 0.05). The fifth placed classifier, fractal analysis of the parallel profile in the upper neck region, was the only one significantly associated with Neck-BMD (*P *= 0.034).

A three-way analysis of variance was used to examine differences in performance due to the region, profile or type of analysis used. It showed that overall PCA analysis performed significantly better than fractal analysis (*P *= 0.019) and that analysis of both the perpendicular and circular profiles performed significantly better than the parallel profile (*P *= 0.003 and 0.011 respectively). No significant differences were found between the different regions of the femoral neck (*P *= 0.241) (despite the apparently large differences in *A*_*z*_). The power of this test was 0.69, 0.97 and 0.15 for the investigation of differences due to the method of analysis, type of profile used and the region analysed respectively.

Table 4 presents the mean A_z _for the slope from each of the spatial frequency bands for all regions of interest. This was assessed for each profile individually and also for all the profiles together. A one-way ANOVA was used to test for significant differences in Az between slopes A, B and C. In the individual profiles, *slopeA *performed significantly better than *slopeC *for the circular profile (*P *= 0.008), however when all the profiles were considered, no significant differences were apparent (*P *= 0.26).

**Table 4:** Comparing *slopeA*, *slopeB *and *slopeC*. The average and standard deviation of the area under the ROC curve (*A*_*z*_) are presented for each of the slopes used in the fractal analysis for all regions of interest. A significant difference was found between *slopeA *and *slopeC *in the circular profile, however when all the profiles were compared, no significant differences were found.

	***SlopeA***	***SlopeB***	***SlopeC***	***P***
All profiles	0.601 (0.074)	0.598 (0.055)	0.565 (0.067)	0.260
Circular	**0.670 (0.072)**	0.611 (0.022)	**0.531 (0.**026)	**0.008**
Parallel	0.544 (0.042)	0.620 (0.083)	0.563 (0.037)	0.140
Perpendicular	0.589 (0.047)	0.563 (0.032)	0.600 (0.104)	0.678

## Discussion and conclusions

In these short series, this study found that texture analysis of standard radiographs using the fast Fourier transform can yield variables that are significantly associated with fracture but not significantly correlated with age, body mass index or Neck-BMD. Both PCA and fractal analysis of the FFT data could be used to discriminate successfully between the groups, although overall PCA was significantly stronger than fractal dimension. The best results from this study were not significantly correlated with femoral neck-BMD, age or BMI, indicating their potential for use as an independent predictor of fracture. The radiographic appearance of bone is known to be affected by factors including the size of the patient. As there was no significant difference in the BMI of the fracture and control groups, it is unlikely that this has influenced the results, however it is an issue that will need addressing in future studies.

**Figure 5:**
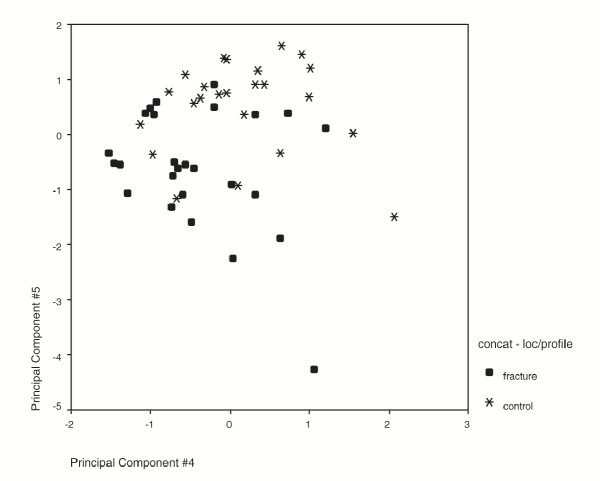
**Plot of two principal components. **Example of a scatterplot of two principal components. For FFT/PCA analysis of the upper head region, principal components 4 and 5 were selected by stepwise analysis and are shown here. They are plotted against each other with fracture and control subjects identified using separate markers. The lack of correlation between the components can be seen (*r *= 0.040, *P *= 0.997).

The PCA method extends a method previously developed for analysis of histological sections [26]. The use of oriented profiles improved the performance of the analysis by selecting directions in which there was the most information about bone structure i.e. perpendicular to the preferred orientation of the trabeculae. PCA considerably reduces the number of variables required to characterise the image via its power spectrum. For example, in this study, we start with a 256 × 256 pixel ROI (65,536 pixels), the Fourier transform is performed and a profile of 128 spatial frequency values is generated. For each profile, PCA was able to describe over 70 % of the variance present in the data using only 5 components or fewer. Overall, the performance of principal components analysis was significantly stronger than that of fractal analysis (*P *< 0.01). One advantage of PCA that may contribute to this finding is the ability to summarise the information present in the dataset with a small number of components via an economical mapping of the variance present in the data. In addition, the property of orthogonality between these components ensures that the variables generated are linearly independent (Fig. 5). Benefits can also be found by the use of a model built on the mathematical distributions present in the data, rather than expecting the data to meet a given mathematical property, such as fitting a fractal distribution.

Previous studies using non-fractal analysis of the Fourier power spectrum have focussed on images of the spine or wrist, where the alignment of trabeculae is generally orthogonal [28-30]. In such images, analysis of trabecular orientation can be performed by examining the vertical and horizontal sectors as the trabeculae lie predominantly in these directions. The trabecular structure of the femur is more complicated as the trabeculae are aligned in arcs, so the preferred orientation changes throughout the proximal femur. Analysis parallel to the preferred orientation of the trabeculae was significantly poorer than analysis using either the perpendicular or circular profiles (*P *< 0.05). Analysis in the perpendicular direction was strongest overall, although it was not significantly better than the circular profile. This accords with the increasingly anisotropic nature of trabecular bone with aging; bone loss is not evenly distributed but is lost primarily at angles perpendicular and oblique to the preferred orientation of the trabeculae [30]. This loss heightens the risk of fracture, especially if the impact is from the side, as expected from a typical fall from standing height, as there are fewer trabeculae orientated in this direction to absorb the force of impact.

In summary, this paper presents a new method for analysing the structure of trabecular bone from standard radiographs. It demonstrates that the Fourier transform can be used to describe structural information in images which may be related to fracture, independently of BMD. This study is limited by the small size of the data set and further analysis is needed to validate these findings. This should be performed on a similar series of radiographs, consisting of fracture and control subjects scanned at the same resolution. The methods from this study could then be applied directly to this group (without recalculating the PCA) to evaluate whether they were generally applicable. However the success of both this and our previous study, using similar techniques to analyse histological sections, indicates that this may be an effective method with clinical utility for describing bone quality statistically in terms of structural parameters.

## Competing interests

The authors declare that they have no competing interests.

## Authors' contributions

Author JG helped design the study, performed the image and data analysis and drafted the manuscript

Author AS collected the data/images used within this study and helped with writing of the paper.

Author PU assisted with some of the practical approaches, and the writing of the paper

Author DMR designed the initial case control study and assisted with interpretation of the results and writing the paper

Author RMA helped with the design of the study, the interpretation of the results and the writing of the paper.

All authors read and approved the final manuscript

## Pre-publication history

The pre-publication history for this paper can be accessed here:


